# Molecular and physiological responses in roots of two full-sib poplars uncover mechanisms that contribute to differences in partial submergence tolerance

**DOI:** 10.1038/s41598-018-30821-y

**Published:** 2018-08-27

**Authors:** YanJie Peng, ZhiXiang Zhou, Zhe Zhang, XiaoLi Yu, XinYe Zhang, KeBing Du

**Affiliations:** 1 College of Horticulture and Forestry Sciences/Hubei Engineering Technology Research Center for Forestry Information, Huazhong Agricultural University, Wuhan, 430070 P. R. China; 2 Hubei Academy of Forestry, Wuhan, 430075 P. R. China

## Abstract

Poplar is a major afforestation tree species in flood-prone areas. Here, we compared molecular and physiological responses in the roots of two full-sib poplar clones, LS1 (flood-tolerant) and LS2 (flood-susceptive), subjected to stagnant flooding using transcript and metabolite profiling. LS1 displayed less phenotypic damage and superior leaf gas exchange and plant growth compared with those of LS2. We concluded that three characteristics might contribute to the differences in flood tolerance between LS1 and LS2. First, fermentation was initiated through lactic dehydrogenation in LS1 roots under flooding and subsequently dominated by alcohol fermentation. However, lactic dehydrogenase was persistently active in flooded LS2. Second, 13 differentially expressed genes associated with energy and O_2_ consumption processes under soil flooding had lower transcript levels in LS1 than those in LS2, which might contribute to better energy-/O_2_-saving abilities and behaviours in flood-tolerant LS1 than those in flood-susceptible LS2 under hypoxic stress. Third, LS1 possessed increased reactive oxygen species scavenging abilities compared with those of LS2 under edaphic flooding. Our data are a valuable contribution to understanding the mechanisms involved in the flood tolerance of poplar.

## Introduction

The growth of plants is usually negatively affected by flooding stress. Because of the characteristics of fast growth and flood tolerance, poplar has served as a major afforestation tree species in flood-prone areas to utilize these flood-affected soils^1^. However, flooding stress still negatively affects poplar growth and survival in these regions^2,3^. Thus, understanding the mechanism involved in the flood tolerance of poplar is important, which may facilitate developing poplar clones with improved flood tolerance.

Plants evolved various mechanisms to withstand flooding stress, including features of morphology and anatomy, metabolism and molecular transcriptional regulation^4–9^. The primary metabolic responses to flooding in plants include the stimulation of fermentative pathways and an increase in glycolytic flux, indicated by elevated transcript abundance, increased activities of pyruvate decarboxylase and alcohol dehydrogenase (ADH), and highly accumulated product (acetaldehyde and ethanol) contents in flooded roots^10,11^. Because of the energy limitation that results from the switch from respiration to fermentation, ammonium and nitrate uptake in roots is also often strongly impaired by flooding^12^. Transcript abundance profiles demonstrate that many genes involved in amino acid biosynthesis and degradation are differentially expressed in flooded trees^13–15^. Many of the amino acids that increase under hypoxia are formed from pyruvate (e.g., leucine, valine and alanine) or other intermediates of glycolysis (e.g., tyrosine, serine and glycine). By contrast, contents of amino acids derived from tricarboxylic (TCA) cycle components (e.g., glutamine, asparagine, glutamate and aspartate) are often lower in hypoxic than in normoxic roots^10,12,13^. Reactive oxygen species (ROS) generating and scavenging systems also play important roles in the ability of plants to respond to various abiotic and biotic stresses^16^. In addition to the role in oxidative damage, ROS also act as signal molecules for adaptive stress responses^17^. In the adaptation of plants to flooding, the group VII Ethylene Response Factor genes (*ERF-VIIs*) play pivotal roles through regulation of anaerobic gene expression and antithetical survival strategies^18^.

Characteristics of morphology, anatomy, eco-physiology and growth of poplar under hypoxia stress are well characterized in the previous literature, accompanied by a few studies on the transcriptome and metabolome^19–21^. Kreuzwieser (2009)^11^ revealed metabolite changes occur in both leaves and roots of flooded grey poplar (*Populus* × *canescens*), but changes in transcript abundance were restricted to the roots. Additionally, increased glycolysis, induced fermentation and inhibited energy-intensive processes are crucial for grey poplar (relatively flood-tolerant) to grow better than *Arabidopsis* (relatively flood-sensitive) under low oxygen conditions. Compared with cotton and *Arabidopsis*, a waterlogging-tolerant poplar clone displayed a different transcriptome under early stages of hypoxia stress^14^. The tolerance to flooding differs not only between species but also between different poplar clones^22^. To date, the mechanism that leads to those differences between poplar clones has been ambiguous. Peng (2013)^22^ clarified that roots play a vital role in flood-tolerance of poplar demonstrated by reciprocal grafting. According to previous reports, the root is the most important organ in response to flooding stress in poplar^13,20,23^. Therefore, molecular and physiological responses in roots of two poplar clones following root hypoxia were compared using transcript and metabolite profiling in this study. The two plant materials (flood-tolerant LS1 and flood-susceptible LS2) were full-sib clones and differed in flood tolerance. Under 15-day partial submergence treatment followed by 3-day recovery, the total biomass of LS1 and LS2 decreased by 6.38% and 32.01%, respectively, compared with their watered control^20^. After partial submergence for 21 days and recovery for 6 days, the reduction in the rate of total biomass for LS1 and LS2 was 24.4% and 59.9%, respectively^23^. We aimed to detect the different mechanisms of the two clones in response to flooding stress and to identify candidate genes involved in flood tolerance that may be used in molecular breeding programmes to improve flood tolerance of poplar.

## Results

### Phenotype, leaf gas exchange, chlorophyll fluorescence and plant growth

**Figure 1:**
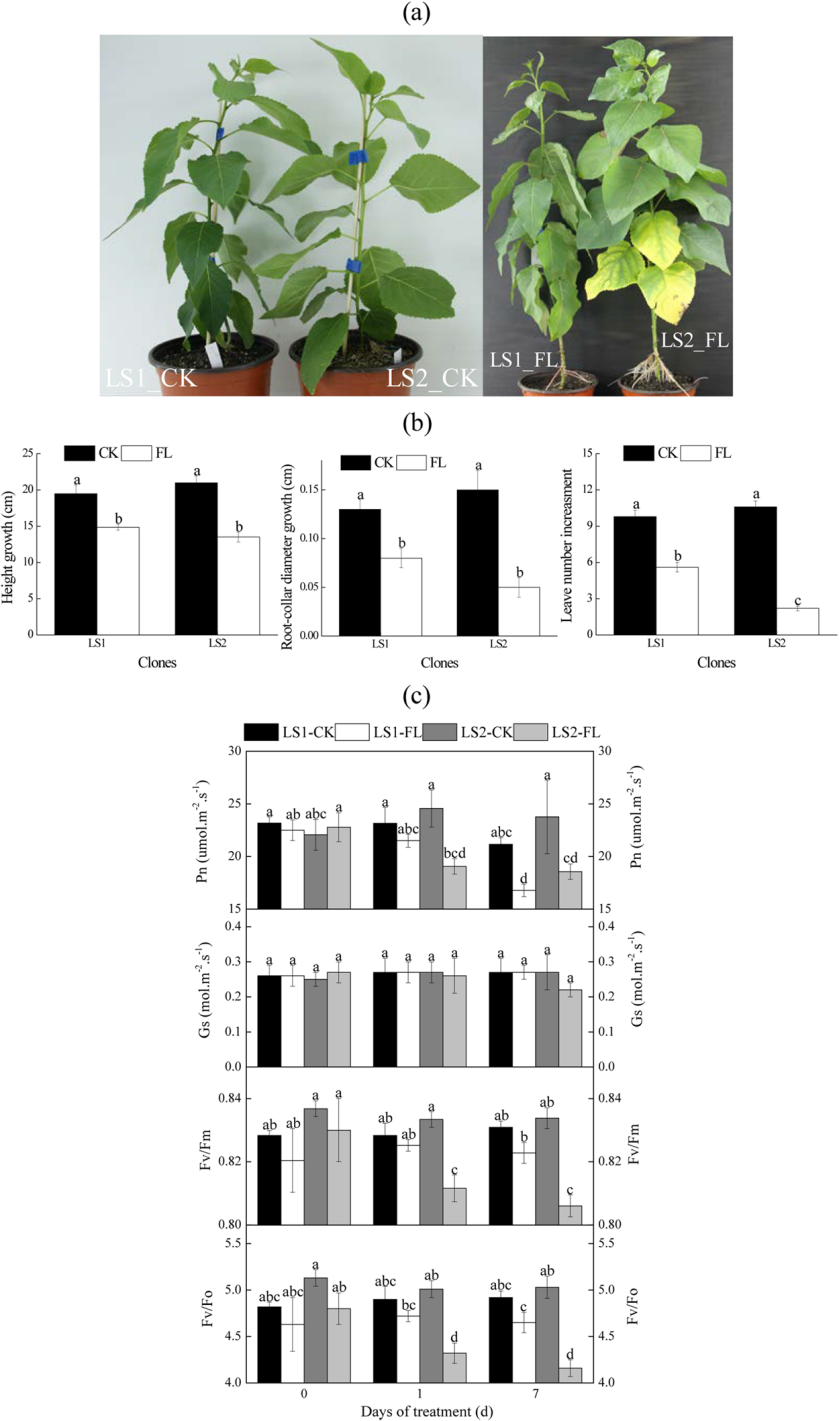
Effects of flooding on phenotype (**a**), growth (**b**), as well as gas exchange and chlorophyll fluorescence (**c**) in LS1 and LS2. The growth parameters were the increase in height, root-collar diameter and leaf number for 18 days during the experiment (from day zero to day 18), respectively. Different letters indicated significant difference at *p* < 0.05 level (Duncan).

Soil flooding negatively influenced the phenotype and most parameters of photosynthesis, chlorophyll fluorescence and growth in both LS1 and LS2 (Fig. 1, Table 1). By the end of the experiment, all plants survived. All watered seedlings grew vigorously without obvious phenotype damage, but for the flooded plants, different levels of phenotype damage occurred between LS1 and LS2 (Fig. 1a). The flooded LS1 grew more new leaves and showed less leaf defoliation than the LS2 during the 18-day treatment (Fig. 1a,b). Height and root-collar diameter growth in the two clones were both dramatically restrained by soil flooding (*p* < 0.05, Fig. 1b). In LS2_FL, net photosynthesis rate (Pn, *p* < 0.05), potential efficiency of primary conversion of light energy of PSII (Fv/Fm, *p* < 0.01) and the ratio of variable fluorescence to initial fluorescence (Fv/Fo, *p* < 0.01) values were significantly lower than those of LS2_CK (Fig. 1c). However, in LS1_FL, non-significant changes were observed with the exception of a decrease in Pn on day 7 (*p* < 0.01, Fig. 1c). Stomatal conductance (Gs) was not noticeably affected by flooding stress in either LS1 or LS2 (Fig. 1c). A reduction in rates due to flooding stress in parameters of leaf gas exchange, chlorophyll fluorescence and plant growth was markedly different (*p* < 0.05) between LS1 and LS2 at every time point, with exceptions of Gs and 7-day Pn. Therefore, LS1 performed better and exhibited more growth than LS2 under soil flooding stress.

**Table 1:** Flooding induced changes in leaf gas exchange, chlorophyll fluorescence and growth in LS1 and LS2.

	LS1_FL1	LS2_FL1	LS1_FL7	LS2_FL7	LS1_FL18	LS2_FL18
Pn	6.15 ± 4.12	21.55 ± 3.33	20.49 ± 2.44	21.17 ± 2.84		
	*p* = 0.019		*p*=0.861			
Gs	−0.45 ± 4.57	−6.10 ± 5.67	−8.02 ± 7.50	18.54 ± 8.83		
	*p* = 0.574		*p* = 0.091			
F_v_/F_m_	0.37 ± 0.42	2.61 ± 0.37	0.99 ± 0.21	3.33 ± 0.33		
	*p* = 0.005		*p* = 0.000			
F_v_/F_o_	3.36 ± 1.59	13.74 ± 1.53	5.49 ± 1.04	17.19 ± 0.66		
	*p* = 0.002		*p* = 0.000			
Height growth					22.90 ± 4.60	35.38 ± 0.28
					*p* = 0.033	
Root-collar diameter growth					39.89 ± 2.18	66.00 ± 6.72
					*p* = 0.006	
Leaf number increase					42.52 ± 4.05	79.44 ± 1.36
					*P* = 0.000	

Note: Rate of changes (%) were displayed by mean ± S.E., *n* = 5; *p* values indicated the significance of the difference between LS1 and LS2 (*t*-test). Pn, net photosynthesis rate; Gs, stomatal conductance; Fv/Fm, potential efficiency of primary conversion of light energy of PSII; Fv/Fo, the ratio of variable fluorescence to initial fluorescence.

### Principal component analysis and gene expression

**Figure 2:**
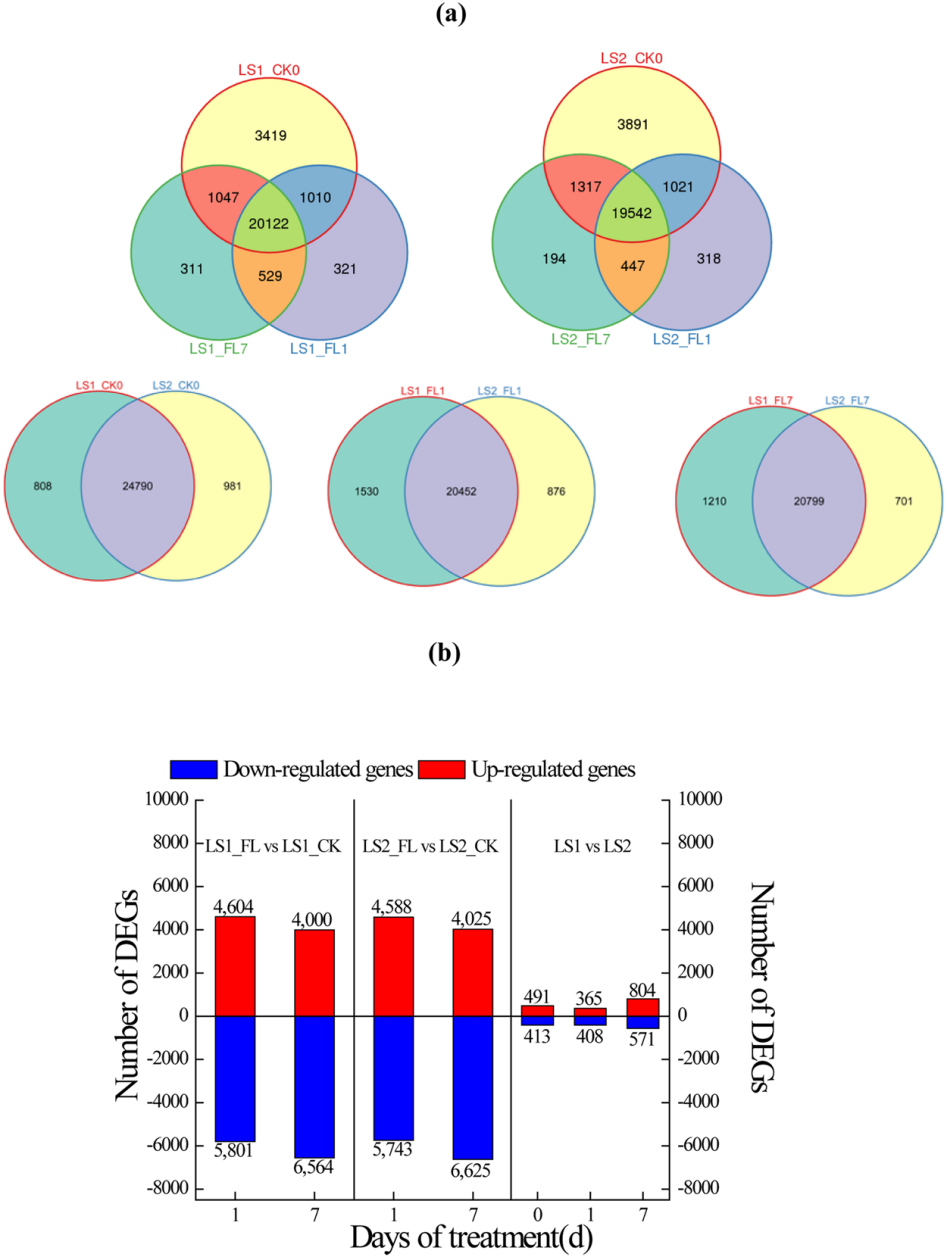
Venn diagram **(a)** and number of DEGs **(b)** between treatment and clones. Venn diagram presented the numbers of treatment- or clone-specific expression genes. DEGs were genes differentially expressed between treatments or clones. Numbers on bars were DEG numbers.

The major characteristics of all libraries are summarized in Supplementary Table 2. Waterlogging stress caused changes in global gene expression levels in both LS1 and LS2 roots (Supplementary Fig. 1). Compared with their controls, a total of 1,161 and 959 genes were only expressed in flooded LS1 and LS2 roots, respectively (Fig. 2a). LS1_FL1 had 74 more differentially expressed genes (DEGs) than LS2_FL1, and LS1_FL7 had 86 fewer DEGs than LS2_FL7 (Fig. 2b). Several clone-specific expression genes (Fig. 2a) and DEGs between LS1 and LS2 were also investigated (Fig. 2b). Compared with LS2, DEG numbers in LS1 on days zero, 1 and 7 were 904, 773 and 1,375, respectively.

### Quantitative real-time polymerase chain reaction validation of the DEGs from the RNA-seq analysis

The correlations between results of RNA-seq and quantitative real-time polymerase chain reaction (qRT-PCR) were evaluated by the expression of the selected 18 DEGs in Supplementary Table 1, including ten transcription factor genes and four genes encoding some important enzymes involved in plant response to abiotic stress. Scatterplots were generated by comparing the log2-fold changes determined by RNA-seq and qRT-PCR in 1-day flooding versus CK and 7-day flooding versus CK in each clone. These transcripts displayed similar expression patterns between qRT-PCR and RNA-seq experiments illustrated by 0.895 and 0.925 correlation coefficients between the two sets of data for LS1 and LS2, respectively (Supplementary Fig. 2). These results confirmed the accuracy and reproducibility of the RNA-seq profiles in this study.

### Functional annotation and classification of DEGs

**Figure 3:**
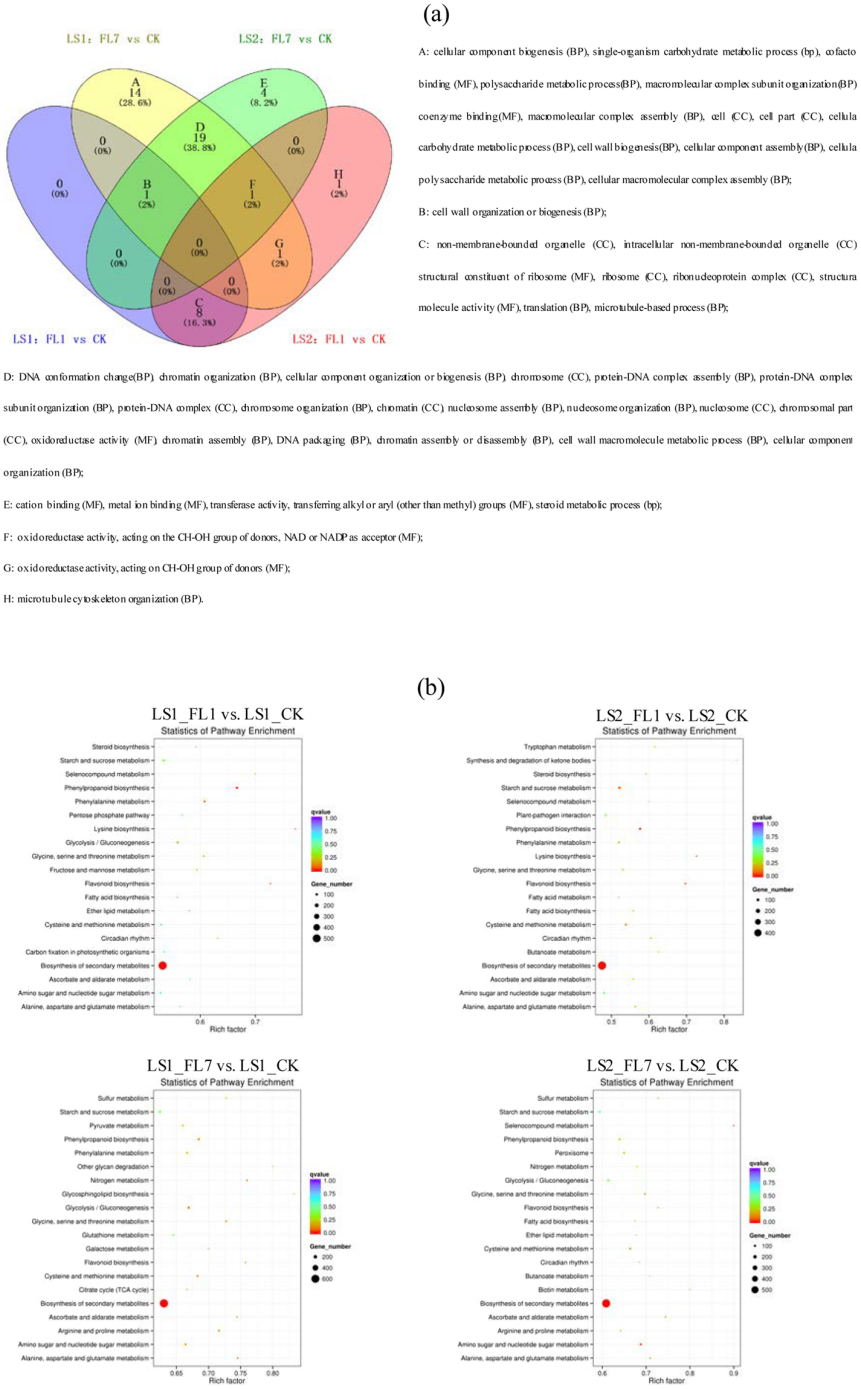
Most enriched GO terms (**a**) and KEGG pathways (**b**) in LS1_FL and LS2_FL compared with CK. Venn diagram of significant enriched GO terms (corrected *p*-value < 0.01) among four conditions were presented. GO terms were classified into three main categories: MF, molecular function; CC, cellular component; BP, biological process. Only top 20 enriched KEGG pathways were showed.

The gene ontology (GO) terms of the unigenes were categorized into molecular functions, cellular components and biological processes. A total of 12,947 unigenes were assigned in the secondary category in accordance with the GO assignments (Supplementary Table 4). Compared with the control, a total of 4,695 (accounting for 36.26% of the 12,947 unigenes), 5,740 (44.33%), 4,190 (32.36%) and 5,515 (42.60%) DEGs in LS1_FL1, LS1_FL7, LS2_FL1 and LS2_FL7, respectively, were assigned to GO terms (Supplementary Table 5). A total number of 49 GO terms were significantly enriched in either LS1 or LS2 under flooding treatment (corrected *p* < 0.05), including 30 GO terms in both (Fig. 3a). Most significantly enriched GO terms had more down-regulated genes than up-regulated genes (Supplementary Table 5). Fourteen GO terms were specifically enriched in LS1_FL7. One and four GO terms were specifically significantly enriched in LS2_FL1 and LS2_FL7, respectively. Cell wall biogenesis, an energy-consuming biological process, was only significantly enriched in LS1_FL7 with 6 up-regulated DEGs and 22 down-regulated DEGs (Supplementary Table 5).

DEGs were also classified into pathways of poplar metabolism based on the Kyoto Encyclopedia of Genes and Genomes (KEGG) database (Fig. 3b; Supplementary Table 6). In total, 9,906 genes were assigned into KEGG pathways; 4,551 (accounting for 45.94% of the 9,906 genes), 5,575 (56.28%), 4,084 (41.23%) and 5,366 (54.17%) DEGs were, respectively, classified into pathways in LS1_FL1, LS1_FL7, LS2_FL1 and LS2_FL7, and the biosynthesis of secondary metabolites was the most enriched pathway. Most enriched pathways in the two clones were classified into carbon metabolism, nitrogen metabolism, lipid metabolism and energy metabolism, including glycolysis, fermentation, fatty acid metabolism and amino acid metabolisms. The top 10 DEGs and corresponding KEGG pathways are listed in Supplementary Table 7.

### Effects on carbon metabolism

**Figure 4:**
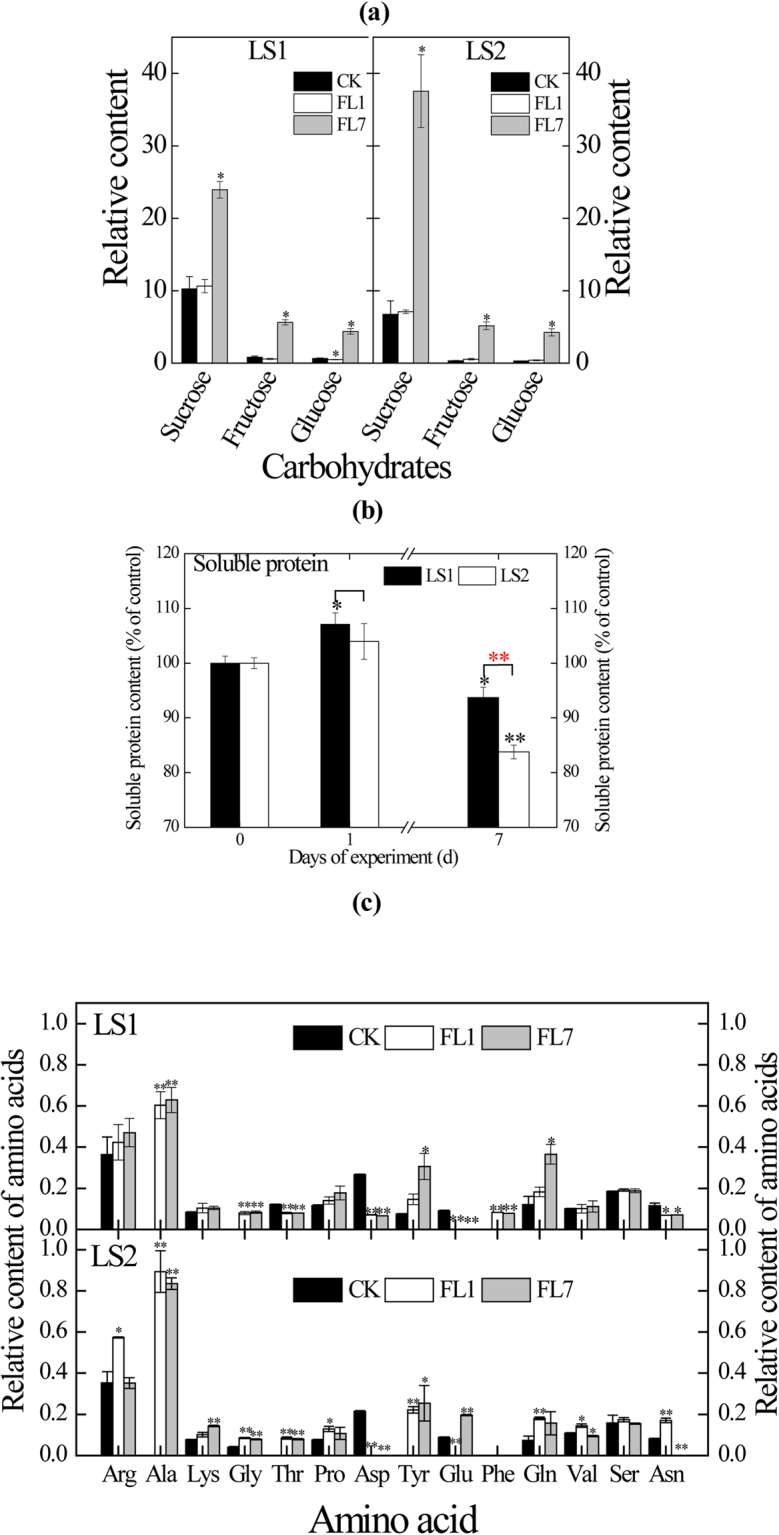
Contents of carbohydrates **(a)**, soluble protein **(b)** and amino acid **(c)** in LS1 and LS2 roots under hypoxia. Carbohydrates and amino acid contents were displayed by relative contents normalized to internal standard (ribitol). Soluble protein content was displayed by % of the control. Statistically significant difference between treatments and controls were indicated by black asterisks, statistically significant difference between LS1 and LS2 were indicated by red asterisks. One asterisks and double asterisks indicated significant difference at *p* < 0.05 and *p* < 0.01 levels, respectively.

**Figure 5:**
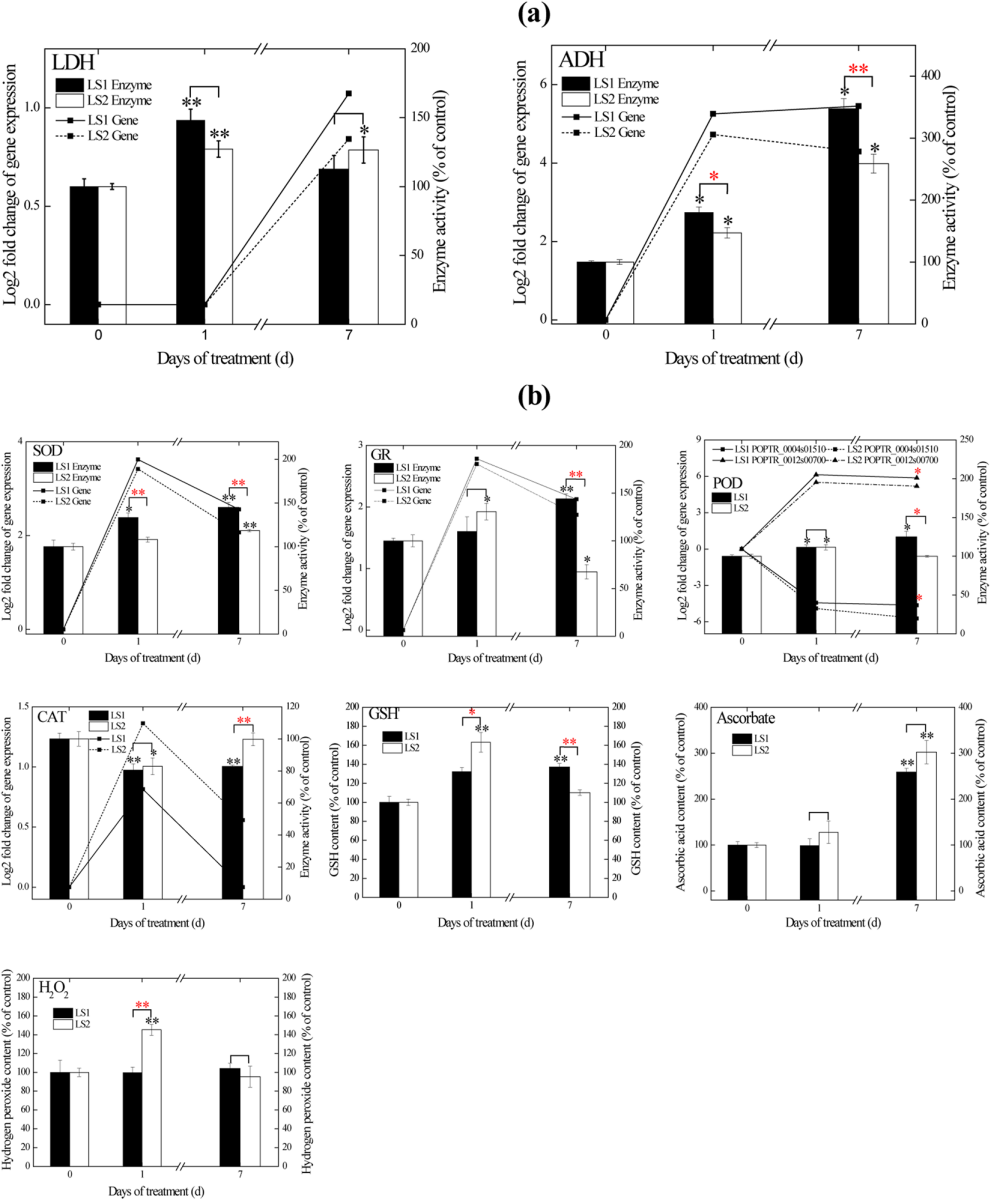
Effects of hypoxia on gene expressions, enzyme activities and substance contents involved in fermentation **(a)** and ROS generation/scavenging system **(b)** in LS1 and LS2 roots. Transcript levels obtained from RNA-seq data were displayed by log2-fold changes compared to the control. Enzyme activities and substance contents were showed by percentage of the control. Statistically significant difference between treatments and controls were indicated by black asterisks, statistically significant difference between LS1 and LS2 were indicated by red asterisks. One asterisks and double asterisks indicated significant difference at *p* < 0.05 and *p* < 0.01 levels, respectively.

Soil flooding caused increased glycolysis, inhibited O_2_-demanding processes (e.g., TCA cycle), and activated alcohol and lactic fermentation in the present study (Supplementary Table 6). Most genes associated with glycolytic processes were up-regulated by hypoxia stress. For example, phosphohexokinase (EC 2.7.1.11) gene *POPTR_0006s25170* and pyruvate kinase (EC 2.7.1.40) gene *POPTR_0008s00310* were up-regulated more than 48-fold and 10-fold, respectively, in LS1_FL and LS2_FL, accompanied with dramatically increased soluble sugar (sucrose, fructose and glucose) contents on day 7 (Fig. 4a). Expression levels of almost half the genes of the TCA cycle were decreased by 1-day flooding and further decreased on day 7. Pyruvate decarboxylase (EC 4.1.1.1) gene *POPTR_0011s07630* and ADH (EC 1.1.1.1) gene *POPTR_0002s07290*, two key genes associated with ethanol fermentation, were activated (>9.5-fold and 19.6-fold, respectively) under flooding. In parallel with the transcript levels, ADH activities increased significantly on the 1st day in both clones (*p* < 0.05) and were highest on the 7th day (*p* < 0.05, Fig. 5a). For lactate dehydrogenase (LDH), activities increased significantly in both LS1_FL and LS2_FL (*p* < 0.05), with the exception of LS1_FL7 (Fig. 5a).

In this study, the sucrose degradation patterns were strongly affected in the roots of LS1_FL and LS2_FL induced by sucrose synthase (EC 2.4.1.13) and invertase (EC 3.2.1.26). For sucrose synthase, *POPTR_0002s20340* (*SUSY3*) was up-regulated more than 21.6-fold on the 1st day and 6-fold on the 7th day in the two clones (Supplementary Table 6). For invertase, one gene *POPTR_0003s11210* was downregulated in both LS1_FL (>27-fold) and LS2_FL (>6.4-fold). Another invertase-encoding gene *POPTR_0006s22710* was up-regulated (>6.6-fold) in LS2_FL but was unchanged in LS1_FL (Supplementary Table 6). Some sucrose transporter genes (*SUT*, *SWEET* and *TMT*) were also differentially expressed between different treatments or clones (Supplementary Table 8).

### Effects on nitrogen metabolism

In comparison with the control, NO_3_^−^ uptake in both LS1_FL and LS2_FL roots was most likely negatively affected as indicated by the 5–6 significantly down-regulated NO_3_^−^ transporter genes and decreased soluble protein contents (Supplementary Table 8; Fig. 4b). Two NO_3_^−^ transporter genes, *POPTR_0015s09290* (*PTNRT2–6*, >5.5-fold) and *POPTR_0015s09310* (*PTRNTR2–3*, >23.6-fold), were upregulated on day 7. Different response levels also occurred between LS1 and LS2. Compared with LS2, LS1 showed four significantly down-regulated transporter genes on day zero and three significantly upregulated transporter genes on day 7 (Supplementary Table 8). For NH_4_^+^ uptake, numbers of transporter genes that showed increased transcript abundance were markedly greater than those of down-regulated transporter genes in both flooded LS1 and LS2 (Supplementary Table 8). Compared with LS2, LS1 showed six significantly up-regulated transporter genes on day 7.

A wide variety of amino acid contents in LS1 and LS2 roots changed markedly during flooding treatment, together with the transcript levels of corresponding genes associated with amino acid metabolism (Supplementary Table 6; Fig. 4c). Most amino acids closely derived from pyruvate (e.g., Ala, Val) and intermediates of glycolysis (e.g., Gly, Tyr) were highly accumulated, whereas contents of most amino acids derived from intermediates of the TCA cycle (e.g., Glu, Asp and Asn) decreased, with the exception of increased Tyr and Val in LS2_FL7 and Asn in LS2_FL1. These results supported the responses of carbon metabolism (i.e., glycolysis and TCA cycle) to flooding in LS1 and LS2 roots at the transcript level.

Different responses to flooding between LS1 and LS2 roots were also observed in amino acid contents (Fig. 4c). For example, in comparison with the controls, Arg content increased non-significantly in both LS1_FL1 and LS1_FL7 but increased significantly in LS2_FL1 (*p* < 0.05) and was unchanged in LS2_FL7. Glu content decreased significantly in both LS1_FL1 and LS1_FL7 (*p* < 0.01), whereas the content significantly decreased in LS2_FL1 (*p* < 0.01) and increased in LS2_FL7 (*p* < 0.01). For Phe content, a significant increase was observed in LS1_FL (*p* < 0.05) but not in LS2_FL.

Different transcript levels of genes involved in amino acid metabolism were also detected between LS1_FL and LS2_FL (Supplementary Table 6). In Arg metabolism, expression of the nitric-oxide synthase (NOS, EC 1.14.13.39) gene *POPTR_0001s00700* was up-regulated in LS2_FL1 (2.4-fold) but uninfluenced in LS1_FL1. In Phe catabolism, transcription of the 4-hydroxyphenylpyruvate dioxygenase (EC 1.13.11.27) gene *POPTR_0002s05840* was up-regulated in LS2_FL roots (5.2-fold and 2.1-fold on the 1st and 7th day, respectively) but was uninfluenced in LS1_FL roots, which explained the response of the Phe contents in the two clones (Fig. 4c).

### Effects on reactive oxygen species generation and scavenging

Soil flooding affected the systems of ROS generation and scavenging in both LS1 and LS2, and the two clones responded differently (Supplementary Table 6; Fig. 5b). For antioxidant enzymes, superoxide dismutase (SOD, EC 1.15.1.1) activities increased in both LS1_FL and LS2_FL, but the increase was significantly greater in LS1_FL (*p* < 0.01). Correspondingly, the SOD gene *POPTR_0013s05350* was also up-regulated in both LS1_FL (>5.9-fold) and LS2_FL (4.2-fold). Peroxidase (POD) activities were markedly elevated in LS1_FL (*p* < 0.05) and LS2_FL1 (*p* < 0.05) but not in LS2_FL7. At the gene transcription level, the POD (EC 1.11.1.7) gene *POPTR_0012s00700* was up-regulated more than 58.4-fold and 36.4-fold in LS1_FL and LS2_FL, respectively. However, another POD gene *POPTR_0004s01510* was downregulated more than 21.6-fold and 30.0-fold in LS1_FL and LS2_FL, respectively. LS2_FL7 displayed higher CAT (EC 1.11.1.6) activity than that of LS1_FL7 (*p* < 0.01). The catalase (CAT) gene *POPTR_0002s01080* was also up-regulated more in LS2 than that in LS1 with a fold change of 2.6, 1.5, 1.8 and 1.1 in LS2_FL1, LS2_FL7, LS1_FL1 and LS1_FL7, respectively. Glutathione reductase (GR, EC 1.8.1.7) activities were, respectively, 109.8% and 144.0% of the control in LS1_FL1 and LS1_FL7 and 130.7% and 67.6% of the control in LS2_FL1 and LS2_FL7. Correspondingly, the GR gene *POPTR_0015s04650* transcript level was up-regulated 6.9-fold, 4.4-fold, 6.5-fold and 3.7-fold. The glutathione peroxidase (GPX, EC 1.11.1.9) gene *POPTR_0003s12620* was up-regulated in both LS1_FL (>1.6-fold) and LS2_FL (>2.0-fold). For non-enzymatic antioxidants, glutathione (GSH) contents were, respectively, 132.1% and 137.2% of the control in LS1_FL1 and LS1_FL7 and 163.1% and 110.1% of the control in LS2_FL1 and LS2_FL7. Ascorbate (Asc) dramatically accumulated in LS1_FL7 (*p* < 0.01, 258.9% of the control) and LS2_FL7 (*p* < 0.01, 302.4% of the control) but not in LS1_FL1 and LS2_FL1. For H_2_O_2_, the content was only significantly elevated in LS2_FL1 (*p* < 0.01, 145.2% of the control) but not in LS1_FL and LS2_FL7.

### DEGs associated with ATP/O_2_ metabolism and transcription factors

**Figure 6:**
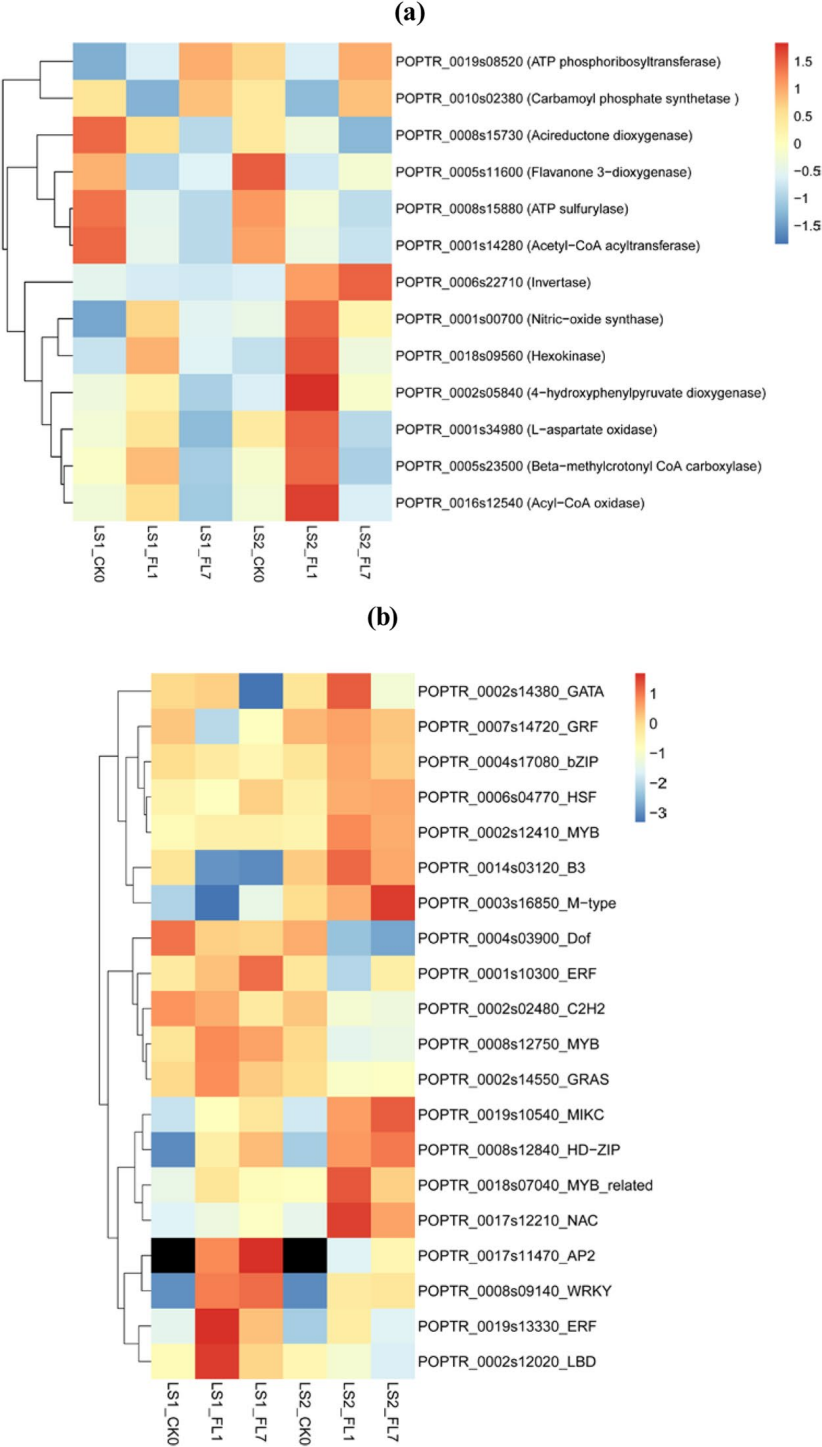
Heatmap of genes involved in ATP or O_2_ consumption **(a)** and transcription factors (**b**) differently expressed between LS1 and LS2 under flooding stress. Log2-fold change values of gene RPKM values were normalized to means before heatmap construction.

Thirteen DEGs associated with ATP/O_2_ metabolism differentially responded to flooding stress between LS1 and LS2 (Fig. 6a), and ATP/O_2_ consumption genes were less expressed in LS1_FL, which might contribute to the difference in flood tolerance. The ATP phosphoribosyltransferase (EC 2.4.2.17) gene *POPTR_0019s08520*, involved in ATP generation, was only up-regulated (2-fold) in LS1_FL7. The other 12 genes involved in O_2_/ATP consumption all had lower transcript levels in LS1 roots than those in LS2 roots under flooding stress, including the invertase gene *POPTR_0006s22710*, 3′-phosphoadenosine 5′-phosphosulfate synthase (EC 2.7.7.4/2.7.1.25) gene *POPTR_0008s15880*, acetyl-CoA acyltransferase 1 gene (EC 2.3.1.16) *POPTR_0001s14280*, acyl-CoA oxidase (ACX) gene *POPTR_0016s12540*, NOS gene *POPTR_0001s00700*, carbamoyl-phosphate synthase gene *POPTR_0010s02380* and 4-hydroxyphenylpyruvate dioxygenase gene *POPTR_0002s05840*, among others.

A total number of 1,038 and 1,006 transcription factors (belonging to 52 families) were differentially expressed in flooded LS1 and LS2 compared with their controls, respectively. *AP2/ERF*, *bHLH*, *MYB*, *NAC* and *bZIP* were the top 5 families that contained the most DEGs in both LS1 and LS2. Expression patterns of several transcription factors differentially responded to flooding stress between LS1 and LS2, and 20 (from 17 families) are listed in Fig. 6b, including *AP2/ERF*, *MYB*, *WRKY*, *NAC*, and *HSF*, among others. The 20 genes were differentially expressed between LS1 and LS2 on both day 1 and day 7 but not on day zero.

## Discussion

In comparison with LS1, LS2 showed more phenotypic damage and greater reductions in values of Pn, F_v_/F_m_, F_v_/F_o_, height growth and root-collar diameter growth under flooding stress (Fig. 1, Table 1), which indicated a more severe flood-injury and an inferior flood tolerance^23,24^. Generally, restrictions on both stomatal (stomatal closure, indicated by Gs) and non-stomatal factors (factors except for stomatal closure, i.e., biochemical reactions of photosynthesis) can result in decreased photosynthesis efficiency under abiotic stress^25^. In our study, Pn values decreased significantly in both flooded LS1 and LS2, whereas their Gs values were reduced non-significantly. The decrease in Pn values might be primarily caused by non-stomatal factors, such as the reduced activity of ribulose-1,5-bisphosphate carboxylase (EC 4.1.1.39), glycolate oxidase (EC 1.1.3.15) and phosphoglycolate phosphatase (EC 3.1.3.18), destruction of chloroplast membrane structure, and inhibition of photosynthetic electron transport and PSII activity^26,27^. A similar result was also reported in long-term flooded (up to 5 d) maize plants (*Zea mays* L.), and soil flooding led to a decrease in photosynthesis and ribulose-1,5-bisphosphate carboxylase activity without significant changes in the values of stomatal conductance^26^.

A global analysis of the transcriptome facilitates the identification of systemic gene expression and regulatory mechanisms for tolerance to a certain stress in a plant^28–30^. The DEGs identified in our study between watered and flooded plants were mostly enriched in glycolysis, fermentation, fatty acid metabolism and amino acid metabolism, consistent with the performance of grey poplar under hypoxia^13^.

The increased glycolysis, stimulated alcohol and lactic fermentation, and inhibited TCA cycle pathways in this study are consistent with the transcript profile responses of rice, *Arabidopsis*, and poplar under hypoxia^31^. Soil flooding stress led to limited carbon entry into the TCA cycle and directed carbon entry into fermentation^13^, which was also indicated by the transcripts of genes involved in these biological processes in this study (Supplementary Table 6). Because roots usually suffer an energy crisis under hypoxia due to insufficient ATP produced by the TCA cycle, to compensate, glycolysis is heightened, and aerobic respiration transforms into fermentation to maintain ATP production^13^. Therefore, higher transcript levels of *pyruvate decarboxylase*, *pyruvate kinase* and *ADH* (Supplementary Table 6), coupled with stronger ADH activity (Fig. 5a), suggested a superior ability of ATP production in LS1_FL than that in LS2_FL, in parallel with their difference in flood tolerance. Different fermentation patterns also occurred in hypoxic LS1 and LS2 roots (Fig. 5a). In LS1_FL, fermentation was initiated by activating lactate fermentation and then quickly dominated by alcohol fermentation, elucidated by the activities of LDH and ADH (Fig. 5a); this process is similar to that in reports with grey poplar^13^, *Arabidopsis*^32^ and maize^33^ and consistent with the pH-stat hypothesis^34^. In LS2_FL, however, lactate fermentation played a sustained role during flooding treatment (Fig. 5a). A more efficient fermentation pathway was previously reported to partly explain the improved waterlogging tolerance of flood-tolerant oak^15^, and excessive lactic dehydrogenation could cause continual cytoplasmic acidification, inhibited glycolysis and irreversible cell injury^34^.

Expression of genes associated with nitrogen metabolism in roots was negatively influenced in both LS1_FL and LS2_FL. The decreased expression levels of NO_3_^−^ transporter genes and significantly reduced soluble protein contents (Fig. 4b) are consistent with previous observations of reduced nitrogen uptake in flooded poplar^12,20^. The internal transport of NO_3_^−^ might be more active, whereas NO_3_^−^ uptake was reduced in roots, as suggested by the upregulated transcript levels of *PTNRT2-6*. *PTNRT2-6* performs a role in the transport of NO_3_^−^ from stored pools, such as vacuoles, to the cytoplasm^35^. *PTRNTR2-3* might also have a similar function to that of *PTNRT2-6*, as suggested by the similar response in expression to root hypoxia. The upregulated NH^4+^ transporter genes could be involved in root internal transport, such as NH^4+^ loading into xylem^14^.

In our study, the contents of a wide variety of amino acids responded rapidly to flooding stress in both LS1 and LS2. Consistent with the results in hypoxic grey poplar^13^ and *Arabidopsis*^36^, the most intensively accumulated amino acids were closely derived from pyruvate (e.g., Ala, Val) and glycolysis intermediates (e.g., Gly, Tyr), whereas the amino acids with the greatest decrease in contents were derived from intermediates of the TCA cycle (e.g., Glu, Asp and Asn). Different responses to flooding also occurred between LS1 and LS2 in amino acid contents and the corresponding transcripts (Supplementary Table 6; Fig. 4c). For example, Arg content and the transcript level of the NOS gene *POPTR_0001s00700* both increased significantly (*p* < 0.05) in LS2_FL1 but not in LS1_FL1 (Fig. 4c, Supplementary Table 6). NOS participates in Arg consumption and nitric oxide (NO) formation^37^. Thus, the increase in *POPTR_0001s00700* expression might be a feedback regulation for Arg accumulation in LS2_FL1. Additionally, increased NOS activity might also lead to increased NO emission in LS2_FL, as reported in several flood-sensitive tree species^38,39^. Compared with that of LS2_FL, Phe was significantly accumulated in LS1_FL (*p* < 0.01, Fig. 4c), and the lower Phe content in LS2_FL might result from the elevated transcript level of *POPTR_0002s05840* in LS2_FL. This gene encodes 4-hydroxyphenylpyruvate dioxygenase, an important enzyme in catalysing Phe catabolism in most plant organs^40^. In comparison with LS1_FL, upregulated *POPTR_0002s05840* expression in LS2_FL would expedite Phe consumption, consistent with the Phe contents observed.

LS1 had stronger ROS-scavenging abilities than those of LS2 under soil flooding in this study, which could contribute to its superior flood tolerance. A wide variety of enzymes and non-enzymatic antioxidants participate in the scavenging of ROS. In enzymatic systems, SOD constitutes the first line of defence against ROS by dismutating O_2_^−^ to H_2_O_2_, and then H_2_O_2_ is decomposed by POD and CAT^41^. Compared with those in LS2_FL, the higher activities of SOD and POD in LS1_FL could contribute to its higher oxidation resistance under hypoxia. As one of the ROS, H_2_O_2_ significantly accumulated in LS2_FL1 (*p* < 0.01, 145.2% of the control) but not in LS1_FL1. The excess H_2_O_2_ could cause severe damage to cell membrane systems and flood-injury in LS2^42^. SOD, L-aspartate oxidase and ACX were pivotal enzymes involved in H_2_O_2_ production in this study^43–46^. The L-aspartate oxidase gene *POPTR_0001s34980* and ACX gene *POPTR_0016s12540* were more upregulated in LS2_FL1 than in LS1_FL1, which might increase the activities of the two enzymes and contribute to the high H_2_O_2_ content in LS2_FL1. POD, CAT, Asc peroxidase, Asc and GSH were key enzymatic/non-enzymatic antioxidants associated with H_2_O_2_ degradation in the two clones^47–49^. In plants, the primary substrate for reductive detoxification of H_2_O_2_ is Asc, and GSH primarily participates in the re-reduction of Asc rather than H_2_O_2_ degradation^50^. In the Asc-GSH cycle, GSH can be oxidized to GSH disulfide and be reduced from GSH disulfide by GR^51^. In our study, GR activity and GSH content both dramatically increased in LS2_FL1 (Fig. 5b). Previous research on the SOD-Asc-GSH cycle highlighted that when SOD activity is low, non-enzymatic antioxidants (e.g., GSH) always play an important role in O_2_^−^ scavenging and induce additional H_2_O_2_ production^51^. Thus, considering the low SOD activity in LS2_FL1, high GSH content most likely contributed to the over-accumulation of H_2_O_2_^51^. On day 7, H_2_O_2_ in flooded LS2 recovered to the control level under the effect of reduced GR activity and GSH content, increased SOD and CAT activities, and increased Asc content (Fig. 5b).

Plants commonly suffer from O_2_ deprivation under waterlogging, and the hypoxic condition can cause an energy crisis and induce down-regulated energy consumption^10,52,53^. We concluded that 13 genes differentially responded to partial submergence between LS1 and LS2, whose products primarily participated in energy/O_2_-related metabolism in amino acid metabolism, sucrose degradation, sulfur reduction, and beta-oxidation of fatty acids, among others. For example, *POPTR_0006s22710* encodes invertase and participates in sucrose degradation. Soil flooding stress can suppress invertase expression and promote sucrose synthase expression, switching to the more energy saving pathway^13^. In our results, the transcript level of one invertase gene *POPTR_0003s11210* showed greater decrease in LS1_FL than that in LS2_FL (*p* < 0.05), and another invertase gene *POPTR_0006s22710* only increased in LS2_FL (*p* < 0.05) but not in LS1_FL (Supplementary Table 6), which would cause higher enzyme activity and more energy consumption in LS2_FL than in LS1_FL. In comparison with LS2_FL, lower transcript levels of two genes (*POPTR_0016s12540* and *POPTR_0001s14280*) associated with beta-oxidation of fatty acids might cause a lower activity of beta-oxidation in LS1_FL roots. Beta-oxidation of fatty acids consists of a series of O_2_-requiring processes located in the peroxisomes of plants^54^. Thus, lower beta-oxidation activity in LS1 would also attenuate O_2_ consumption under hypoxia. Other genes listed in Fig. 6a also increased or were not inhibited in flooded LS2 roots, which might lead to over-consumption of energy and O_2_, resulting in severe energy starvation and O_2_ deficiency. By contrast, those genes involved in energy/O_2_ consumption were inhibited in flooded LS1 and contributed to maintaining available energy and O_2_ under hypoxic conditions.

Years of studies on biotic and abiotic stresses show that transcription factors play very important roles in regulating plant stress resistance. Members of families *AP2/ERF*, *WRKY*, *NAC* and *HSF* participate in regulating flood tolerance of plants^27,55–58^. In our study, 20 transcription factors were identified that were differentially expressed between flooded LS1 and LS2, including *AP2/ERF*, *MYB*, *WRKY*, *NAC*, *HSF*, *GATA*, *GRF*, *bZIP*, *B3*, *M-type*, *Dof*, *C2H2*, *GRAS*, *MIKC*, *HD-ZIP*, *MYB_related* and *LBD* family genes (Fig. 6b). Family *AP2/ERF* was well characterized, and abundant *AP2/ERF* family genes participate in regulating flood tolerance in rice, *Arabidopsis* and soybean, such as *Sub1A*, *AtERF73/HRE1*, *SNORKEL*, and *OsEATB*, among others^4,5,59–62^. Hypoxia induction of *ADH* expression levels was reduced in three *AtERF73/HRE1* knockout *Arabidopsis* lines, whereas the expression of *ADH* increased in the *AtERF73/HRE1* overexpression line, which suggested that the *Arabidopsis* gene *AtERF73/HRE1* plays a central role in anaerobic respiration regulation under hypoxia^60^. Expression of rice genes *SNORKEL1* and *SNORKEL2* can be induced by ethylene accumulation under a submerged condition, and products of the two genes then triggered remarkable internode elongation via gibberellin, allowing rice to grow in flooding terrain^61^. In this study, three DEGs, *POPTR_0001s10300*, *POPTR_0017s11470* and *POPTR_0019s13330*, were also classified into family *AP2/ERF* and might also play essential roles in poplar responding to root hypoxia stress. Further work is required to determine whether these DEGs regulate flood tolerance of poplar.

## Materials and Methods

### Plant materials and growth conditions

**Figure 7:**
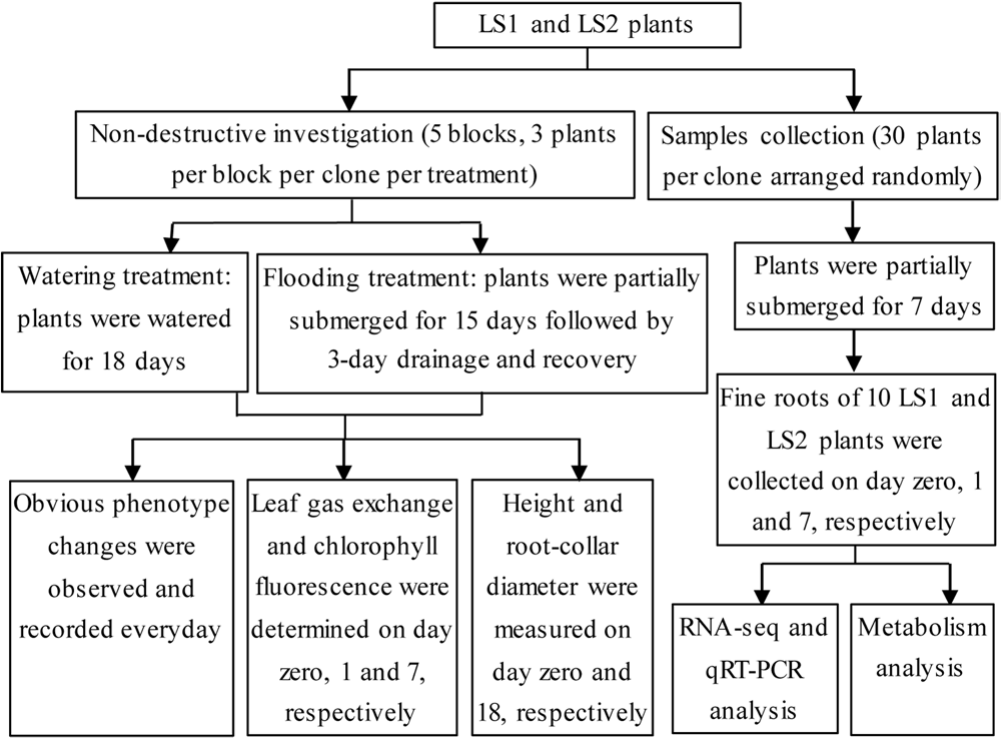
Experiment design drawing of the study.

Two full-sib clones, LS1 (flood-tolerant) and LS2 (flood-susceptible), originating from *P. deltoides* cv. *Lux* ex. *I-69/55* (flood-tolerant) × *P. simonii* (flood-susceptible) were used as the materials in the present study^1^. Four-week-old stem saplings of LS1 and LS2 were transferred from sterile agar cultures into plastic pots (14 cm × 10 cm × 13 cm) containing a mixture of vermiculite and culture soil (1:3, v/v). The soil (pH 6.0) consisted of 2–5% N, P_2_O_5_ and K_2_O, with more than 20% organic matter (dry weight). After establishing growth after transplantation, all plants were watered with Hoagland solution weekly and tap water twice a week. All plants were grown in a room with a temperature of 25 ± 2 °C under a photoperiod of 16 h light and 8 h dark with a light intensity of 40 μmol m^−2^ s^−1^ provided by cool white fluorescent lamps and with 70–80% humidity. All plants with a height of 35–40 cm were randomly assigned to one of two water regime treatments: (1) watered (control check, CK) and (2) flooded (FL). The plants were divided into the two groups, and the experimental design is illustrated in Fig. 7. Pots of the control group had four drainage holes in the bottom. The plants were watered using tap water daily to maintain soil moisture at field capacity. The flooded plants were partially submerged by stagnant flooding to a height of 10 cm above the soil surface in tanks^63^.

After 0, 1, and 7 d of stagnant flooding treatment, 10 trees per treatment per clone were harvested. Fine roots were immediately frozen in liquid nitrogen and kept at −80 °C until utilization for RNA-sequencing (RNA-seq) and metabolism analysis.

For the convenience of description, we defined watered LS1, 1-day flooded LS1, 7-day flooded LS1, and 15-day flooded LS1 followed by 3-day recovery as LS1_CK, LS1_FL1, LS1_FL7 and LS1_FL18, respectively. In LS2, the corresponding definitions were LS2_CK, LS2_FL1, LS2_FL7 and LS2_FL18, respectively. LS1_FL1 and LS1_FL7 composed LS1_FL, and LS2_FL1 and LS2_FL7 composed LS2_FL.

### Measurement of leaf gas exchange and chlorophyll fluorescence

The 5th fully expanded and mature leaf from the top of the stem was chosen to measure leaf gas exchange and chlorophyll fluorescence^23^. Five plants per treatment per clone were measured between 9:00 am. and 11:00 am. using an LI-6400 photosynthesis system (LI-COR Inc., Lincoln, NE, USA) with a standard LI-COR gas exchange chamber. A 1500 μmol m^−2^ s^−1^ light intensity of illumination was provided by red diodes (6400–02 LED Source), and the gas flow rate was set as 500 μmol s^−1^. The gas exchange measurements included Pn and Gs with transpiration rate, intercellular CO_2_ concentration, atmospheric CO_2_ concentration and the corresponding ambient environmental conditions, such as temperature, relative humidity and photosynthetically active radiation. Chlorophyll fluorescence (Fv/Fm and Fv/Fo) of leaves of five plants per treatment per clone was measured using an LI-6400 fluorescence system (LI-COR Inc., USA) after a 20-min dark adaptation. To compare the effects of flooding treatment on plants, changes in all growth and ecophysiological parameters under flooding were calculated as follow: change rate = (CK − FL)/CK, where CK represents the value of the control, and FL represents the value in the flooding treatment.

### RNA-sequencing

Total RNA was extracted and purified using Trizol (Invitrogen, Canada), and 3 μg of purified RNA per sample was used for library construction. Briefly, the first-strand cDNA was synthesized using a random hexamer primer and M-MuLV reverse transcriptase (RNase H); the second-strand cDNA synthesis was subsequently performed using DNA polymerase I and RNase H; and the cDNA fragments were ligated with adapters. These products were purified and amplified by PCR to create the final cDNA library. The clustering of the index-coded samples was performed on a cBot cluster generation system using TruSeq SR cluster kit v3-cBot-HS (Illumina) according to the manufacturer’s instructions. After cluster generation, the library preparations were sequenced on an Illumina Hiseq 2000 platform, and 100 bp single-end reads were generated (Novogene Bioinformatics Institute, China). Two biological replicates per clone per treatment were used in this study, and each biological replicate included five well-mixed individual plants.

### RNA-seq data analysis

Raw data were utilized to obtain clean data by removing reads containing adapter sequences, reads containing poly-N and low quality reads. All the downstream analyses were performed based on the clean data. The genome of *P. trichocarpa*^64^ was used as the reference genome for sequence read alignment and identification. For unigenes, Bowtie v0.12.9 was used to align single-end clean reads to the unigene sequences. HTSeq v0.5.4p3 was used to count the read numbers mapped to each gene. Then, the reads per kilobase of exon model per million mapped reads (RPKM) of each gene was calculated based on the length of the gene and read counts mapped to the gene^65^. In this study, we defined genes with RPKM ≥1 as expressed genes. Principal component analysis was performed using RPKM of 12 RNA-seq libraries in the SPSS 19 statistical software package (IBM Co., USA).

Differential expression analysis of the two conditions was performed using the DESeq R package (1.10.1; TNLIST, China)^66^. The resulting *p*-values were adjusted using the Benjamini and Hochberg’s approach for controlling the false discovery rate^67^. Genes with an adjusted *p*-value (padj) <0.05 and |log2-fold change| ≥1 were assigned as differentially expressed genes (DEGs). Gene ontology (GO) enrichment analysis of the DEGs was implemented by the GOseq R package in which gene length bias was corrected. GO terms with a corrected *p*-value < 0.01 were considered significantly enriched by DEGs. KOBAS software (KOBAS, UK) was used to test the statistical enrichment of DEGs in KEGG pathways.

### Validation by qRT-PCR

Eighteen DEGs were selected to validate all gene expression results from RNA-seq by qRT-PCR (Supplementary Table 1). Five independent biological repeats were conducted, and RNA was reverse transcribed using Primescript^TM^ RT master mix reagent (Takara, China). The qRT-PCR was performed using SYBR^®^ Premix Ex Taq™ II (Takara, China) on a 7500 fast real-time PCR system (Applied Biosystems, NY). Three technical replicates for each gene in all samples were used in qRT-PCR. Some primers were obtained from the literature (Supplementary Table 1), and the others were designed using Premier 5.0 (Premier Biosoft, CA). 18 S rRNA was used as an internal control to normalize all data^68^, and the relative expression of a gene pair was calculated using the 2^−ΔΔ*C*T^ method and presented by log2-fold change values^69^.

### Gas chromatography-mass spectrometer analysis

The same samples used for RNA-seq were also used for gas chromatography-mass spectrometer (GC-MS) analysis. Metabolites from poplar roots (100 ± 10 mg) were extracted, derived and profiled according to the method used in barley^70^ with some modifications. Data were analysed using the R software platform (http://cran.r-project.org/) and Xcalibur software. Identification of compounds was based on a comparison of their mass spectra, retention indices (RIs) and retention times with the authentic standards and published data, in addition to the standard mass spectra published in the NIST2014^71^. Ribitol was used as an internal standard for semi-quantification analysis. By comparing the GC-peak area of each compound with that of the internal standard, relative units were used to express the contents of the metabolites^72^.

### Measurement of biochemical parameters

Measurements of physiological-biochemical parameters of fine roots were conducted (four replicates per treatment per clone) to clarify the metabolisms of energy and ROS, two important pathways that respond to abiotic stress in plants. Tissue homogenate was prepared by grinding 0.5 g of fine roots in 4.5 ml of normal saline. The parameters were measured according to the instruction manuals of the reagent kits (Nanjing Jiancheng Bioengineering Institute, China), including contents of soluble protein (Bradford method), GSH (DTNB method) and H_2_O_2_ and activities of CAT, GR, POD, SOD (Xanthine oxidase method), ADH and LDH. H_2_O_2_ contents were determined spectrophotometrically by monitoring the amount of complex compound (H_2_O_2_ and molybdic acid) at a wavelength of 405 nm. CAT activity was measured by monitoring the decrease in the amount of complex compound due to H_2_O_2_ decomposition at 405 nm. GR activity was determined by detecting the oxidation of nicotinamide adenine dinucleotide phosphate (NADPH) at 340 nm in the presence of glutathione disulfide and expressed as nmol NADPH oxidized per mg of protein per minute. POD activity was measured following the principle described by Montavon^73^. ADH activity was detected spectrophotometrically at 340 nm based on nicotinamide adenine dinucleotide (NAD+) reduction catalysed by ADH. In the LDH activity measurement, LDH in tissue homogenate was used to catalyse pyruvic acid generation. Then, pyruvic acid content was measured by the 2,4-dinitrophenylhydrazine method using an automatic microplate reader (Infinite M200, Austria). LDH activity was finally calculated from pyruvic acid production.

### Statistical analyses

The data on gas exchange, chlorophyll fluorescence and growth were subjected to analysis of variance (ANOVA). The data of enzyme activities, metabolites of ROS and soluble protein were subjected to Student’s *t*-test between flooding treatment and control and between LS1 and LS2 at every time point. The data of carbohydrates and amino acids were subjected to Student’s *t*-test between flooding treatment and control at every time point. All analyses were conducted using the SAS statistical software package version 9.0 (SAS Institute Inc., USA).

### Accession code

Sequence reads of transcriptome sequencing have been deposited in the NCBI sequence read archive under accession number SRP127249.

## Electronic supplementary material

Supplementary Figures 1 and 2

Dataset 1

Dataset 2

Dataset 3

Dataset 4

Dataset 5

Dataset 6

Dataset 7

Dataset 8
