# Supplementary material for: Molecular and physiological responses in roots of two full-sib poplars uncover mechanisms that contribute to differences in partial submergence tolerance
Source: Sci Rep. 2018 Aug 27;8:12829. doi: 10.1038/s41598-018-30821-y (PMC6110812; doi:10.1038/s41598-018-30821-y)
Supplement: Supplementary file 1 — Supplementary Figures 1 and 2 [file 41598_2018_30821_MOESM1_ESM.doc]

Supplementary Material

# Molecular and physiological responses in roots of two full-sib poplars uncover mechanisms that contribute to differences in partial submergence tolerance

YanJie Peng, ZhiXiang Zhou, Zhe Zhang, XiaoLi Yu, XinYe Zhang, KeBing Du*

***Correspondence:** KeBing Du: kebingdu@mail.hzau.edu.cn

**Supplementary Figure 1** Principal component analysis of 12 RNA-seq libraries. RPKM values were used in this analysis.

**Supplementary Figure 2** Correlation of the differentially expressed genes as determined by RNA-seq and qRT-PCR analyses. Changes of the transcript levels of the 18 selected genes were determined by qRT-PCR (primer sets see Supplementary Table S1) in roots of LS1 and LS2, respectively, after different times of flooding stress. The results were plotted against data obtained by RNA-seq and a linear regression was calculated (results presented in graph). Dots were created by log2-fold change values.
